# Associations between Sleep, Depression, and Cognitive Performance in Adolescence

**DOI:** 10.3390/ejihpe13020038

**Published:** 2023-02-18

**Authors:** Raúl Quevedo-Blasco, Amparo Díaz-Román, Víctor J. Quevedo-Blasco

**Affiliations:** 1 Center for Mind, Brain, and Behaviour Research (CIMCYC), University of Granada, 18011 Granada, Spain; 2 Faculty of Psychology, University of Granada, 18011 Granada, Spain; 3 Consejería de Desarrollo Educativo y Formación Profesional, Junta de Andalucía, 18010 Granada, Spain

**Keywords:** daytime sleepiness, sleep quality, cognitive functioning, inductive reasoning, reading comprehension, mathematical thinking, problem-solving, number and calculation, youths

## Abstract

The relevance of cognitive performance during adolescence requires further studies that analyze potential associated factors. This study aimed to analyze inductive reasoning, reading comprehension, and mathematical thinking (problem-solving and number and calculation) in relation to sleep and depression in 244 students aged 12–17 years (47.6% boys and 52.4% girls). Daytime sleepiness, sleep quality, dysthymia, and euthymia (state and trait) were assessed by self-reported questionnaires. Moreover, correlations between these variables and cognitive performance, and differences depending on sociodemographic variables (sex, age, or academic year) were analyzed using non-parametric tests. Robust regression models were also conducted to evaluate the predictive role of significant variables on cognitive performance. The results showed significant bidirectional relationships between sleep- and depression-related variables, and between the latter ones and cognitive performance. Depression-trait was more related to cognitive performance than depression-state, and euthymia more than dysthymia, but neither daytime sleepiness nor sleep quality significantly correlated with it. As for sociodemographic variables, girls reported worse sleep and more depressive symptoms than boys did, and younger students reported better sleep but performed worse than the older ones. Although these findings should be further explored in forthcoming studies adding other promising variables, they highlight the importance of promoting euthymia to improve cognitive performance in adolescents.

## 1. Introduction

For decades, the interest in identifying and analyzing the factors linked to cognitive performance in adolescents has been growing in all fields, and specifically in psychology in the clinical and educational context. This topic has become relevant due to the impact of COVID-19 lockdown on adolescents’ cognitive and academic performance [1,2,3,4]. Although it is true that there are studies on cognitive and executive functions, they commonly relate to adults [5] and more specifically to elderly people [6,7,8,9,10,11]. Other studies have focused on the mediating role of sleep disturbances, anxiety, and depression in people with certain disorders, such as autism [12], epilepsy [13], attention-deficit/hyperactivity disorder (ADHD) [14,15], or schizophrenia [16,17].

That adolescents hardly present mental problems compared to adults is a biased view. Approximately 13–14% of this population presents some mental disorder worldwide, the most incidental and prevalent being those linked to depression and anxiety [18,19]. The depression–adolescence link is not recent; the United Nations has reported on it for years [20]. In 2017, the World Health Organization (WHO) indicated that the second cause of death among young people was associated with depression [21].

The influence of sociocultural and family factors on variables related to anxiety, depression, and performance in youth populations is clear [22,23,24,25,26,27] and the variables include motivational issues as well [28,29]. In this sense, the relationship with other aspects of the own person is also evident, such as emotional regulation [30,31], and with other factors, such as those related to the teaching-learning process itself [32,33].

Concerning sleep, it is evident that sleep quality impacts multiple functions, both physical [34] and cognitive [35,36], and specifically, on the academic performance of young students (e.g., [37]). Even sleep disturbances may have negative side-effects, such as the ones leading to the recurrent appearance of depressive symptoms over the years [38]. In children, it has also been confirmed, to a greater or lesser extent, that minors who have poorer sleep quality obtain lower scores on cognitive tasks [39]. One should not forget the multiple factors that also directly affect sleep (see, for example [40]).

The aim of this study was to analyze the potential relationship between sleep, depression, and cognitive performance in adolescents. To this end, sleep quality and daytime sleepiness were assessed in a sample of adolescents, paying attention to their possible depressive symptoms. In particular, both the depression-state (depressive symptoms presented at the time of assessment) and the depression-trait (depressive symptoms displayed over time) were taken into account. Finally, the possible influence of sleep and depression on cognitive performance was examined, considering three domains involved in academic achievement: inductive reasoning, reading comprehension, and mathematical thinking.

## 2. Materials and Methods

### 2.1. Participants

The initial sample comprised 296 students (141 boys and 155 girls; 47.6% and 52.4%, respectively) recruited through a cross-sectional probability sampling. After deleting those participants excluded due to lack of information or being missing cases (including two adolescents aged 18), a total of 244 students (53.3% girls) were finally analyzed.

### 2.2. Design and Procedure

This is a descriptive study of populations through survey research, with cross-sectional probability sampling (see [41]). The instruments were administered in groups (using pencil and paper), and anonymously, to prevent possible response bias. The students voluntarily completed the assessment tasks. The study was conducted in accordance with the Declaration of Helsinki and was approved by the Institutional Review Board.

Before administering the instruments and to appropriately access the sample, the head of the educational center was provided with two files: (a) information on the study the students would be provided with, and (b) informed consent. The head gave the students these files to be reviewed and signed by their parents or legal tutors. The file containing information on the study described what participation in the study consisted of, and the fact that the study did not lead to undesirable side effects for participants’ health. The informed consent explained, among other issues, the explicit right of every participant to drop out of the study and pertinent confidentiality and data protection guarantee in accordance with the current national and European regulations. It was an essential requirement for those students who had agreed to be evaluated in this research to deliver the informed consent signed by the person in charge.

Beyond the fulfillment and the signature of the informed consent, the students also had to meet the following conditions to take part in the study: being enrolled in compulsory secondary education; not presenting diagnosed psychological disorders; and not being under medication with effects on sleep. To recruit participants, the teachers discussed the choice of participating in their classes. The rejection rate was around 5%. Nine students were ruled out because they were taking sleeping pills and, as mentioned earlier, 52 student records had to be deleted due to missing information. For the completion of the assessment instruments, all the students received the same initial general information and had maximum time for doing it. Emerging doubts were resolved during the performance of the tasks which were conducted in four sessions in groups of 15 students, approximately.

### 2.3. Measures

#### 2.3.1. Sociodemographic Data

An ad hoc questionnaire was administered to collect data on participants’ age, sex (male/boy, female/girl, other, or prefer not to answer), academic year (1st year, 2nd year, 3rd year, or 4th year), and repeater or not repeater condition (yes/no).

#### 2.3.2. Sleep-Related Variables

The Epworth Sleepiness Scale (ESS [42]) and the Pittsburgh Sleep Quality Index (PSQI [43]) were used to assess participants’ daytime sleepiness and sleep quality, respectively. Both scales are self-administered. In the ESS, participants are asked to rate from 0 to 3 their risk of falling asleep in eight daily situations (i.e., reading, watching TV, driving, etc.). The PSQI comprises 19 main items that assess seven sleep components: subjective sleep quality, sleep latency, sleep duration, sleep efficiency, sleep disturbances, use of sleeping medication, and daytime dysfunction. The sum of scores for these seven components yields one global score ranged from 0 to 21 (higher global score indicates worse sleep quality). The participants’ PSQI global score was the measure used in this study. The ESS and the PSQI show appropriate psychometric properties to be employed in the Spanish sample population (Cronbach’s alpha > 0.9 and 0.81, respectively) [44,45].

#### 2.3.3. Depression-Related Variables

The Spanish adaptation of the State-Trait Depression Inventory (STDI [46]) was employed to assess the presence of depression or negative affect (dysthymia), and the absence of depression or positive affect (euthymia) in participants. This questionnaire enables one to assess both the affectation level at the time of assessment (dysthymia- and euthymia-state) and the frequency of occurrence (dysthymia- and euthymia-trait) through 20 items scored on a 4-point Likert scale. Dysthymia, euthymia, and total direct scores are later transformed into percentiles. The questionnaire is adequate to be used both in clinical and non-clinical samples [47]. The total reliability coefficients (Cronbach’s alpha) range between 0.85 and 0.90 (for state) and 0.74 and 0.84 (for trait) in adolescents [46].

#### 2.3.4. Cognitive Performance

Participants’ cognitive performance was assessed with the Psychopedagogical Battery EVALUA [48,49], which is useful to assess both general and specific cognitive domains. The battery is divided into 11 levels or areas of application: from EVALUA-0 (third year of early childhood education) to EVALUA-10 (fourth year of compulsory secondary education). The last version of the instrument is the 4.0. Due to the characteristics of the sample of this study, we used the batteries 7, 8, 9, and 10. Direct scores—transformed into universal percentiles—were obtained for each participant in three domains: inductive reasoning, reading comprehension, and mathematical thinking. Two separate measures of mathematical thinking were computed: problem-solving and number and calculation. The instrument showed appropriate psychometric properties in a sample of 60,000 students (see [48,49]), with reliability coefficients (Cronbach’s alpha) ranged between 0.85 and 0.89.

### 2.4. Statistical Analysis

First, descriptive analyses for every variable were conducted. Second, Spearman correlations coefficients (*r*_s_) were calculated to assess associations between sleep- and depression-related variables and the participants’ performance in inductive reasoning, reading comprehension, and mathematical thinking (problem-solving and number and calculation). Correlations between the previous variables and participants’ age were also computed. Differences in those variables depending on participants’ sex and academic year were analyzed through Wilcoxon rank-sum and Kruskall–Wallis H tests, respectively, using Rosenthal’s *r* as the effect size measure. Afterwards and based on the results of our preceding analyses, parameters of several multiple regression models were estimated by using robust methods, and including sleep, depression, and sociodemographic variables as predictors of scores in each cognitive domain. The *Rstudio 2.5.2.* software was used to conduct data analyses, although we used the *IBM SPSS Statistics 21* to conduct post-hoc pairwise comparisons after the Kruskall–Wallis H test. The statistical significance was set at *p* < 0.05. We opted for non-parametric tests after checking with the help of the Shapiro–Wilk test that data did not meet the parametric assumptions.

## 3. Results

The participants were aged 12–17 (mean age = 14.32, *SD* = 1.33) and were enrolled in compulsory secondary education in a center in Andalusia (Spain): 19.7% in the first year, 25% in the second one, 32% in the third one, and 23.4% in the fourth year. Less than a fourth of the students (14.8%) were repeaters. Regarding their socioeconomic status, they all belonged to a medium level.

### 3.1. Associations between Sleep, Depression, and Cognitive Performance Variables

Neither daytime sleepiness nor sleep quality reported by participants were significantly correlated with their performance percentile scores in any of the three cognitive domains assessed: inductive reasoning, reading comprehension, and mathematical thinking. Participants’ daytime sleepiness was correlated with euthymia-state and dysthymia-state and trait percentile scores. Participants’ sleep quality was significantly correlated with all the depression-related variables, except for state and trait total percentile scores. The significant correlations found between sleep- and depression-related variables were negative for euthymia scores and positive for dysthymia scores (Table 1).

Correlations between participants’ state percentile scores and cognitive performance were not statistically significant, except for dysthymia-state and reading comprehension. However, participants’ trait percentile scores were significantly correlated with all the cognitive performance measures. The significant correlations between cognitive performance and depression-related variables were positive for euthymia-trait and trait total score and negative for dysthymia-trait (Table 1).

### 3.2. Differences in Sleep, Depression, and Cognitive Performance, Depending on Sociodemographic Variables

Correlations between participants’ age and sleep- and depression-related variables were statistically significant for both daytime sleepiness and sleep quality, but they were not so for dysthymia or euthymia, or state or trait total scores. Participants’ age was also negatively correlated with their percentile scores in inductive reasoning and reading comprehension (Table 1).

Participants’ mean scores and standard deviations in sleep- and depression-related variables and cognitive performance measures, depending on sex and academic year, are reported in Table 2 and Table 3.

Girls reported higher daytime sleepiness, worse sleep quality, and lower presence of positive affect and more depressive symptoms—at the time of assessment and in general—than boys did, although they did not significantly differ in terms of cognitive performance (Table 2).

First-year students reported significantly lower daytime sleepiness, higher sleep quality, and higher euthymia-state in comparison with third- and fourth-year students (Table 3). Second-year students also reported lower daytime sleepiness than third- and fourth-year students did, and higher sleep quality (lower PSQI total score) than that of the latter. In contrast, cognitive performance was better in higher grades. Fourth-year students showed significantly higher number and calculation percentile scores than first-year students did, and third-year students scored higher in number and calculation than both first- and second-year students did. The problem-solving performance of the third-year students was significantly better than those of the students in the other grades (Table 3).

### 3.3. Robust Regression Models

Based on the previously mentioned significant results, the variables considered as potential predictors of students’ scores in cognitive domains were euthymia-trait, daytime sleepiness, sleep quality, age, and sex. However, only those variables whose inclusion in each regression model led to an increase higher than 1% in the proportion of explained variance were finally included as predictors for the cognitive domain at issue. Similarly, the variable age was exchanged for the variable educational stage, depending on the proportion of explained variance for each cognitive domain. This latter variable was obtained from the transformation of the 4-level variable academic year into a 2-level variable coded as 0 (first- and second-year) and 1 (third- and fourth-year). The proportions of explained variance by the final robust regression models conducted for each cognitive domain were—based on multiple *R*^2^ values—as follows: 9.39 for inductive reasoning (70.40 + 0.26_euthymia_–_trait_ − 3.46_age_ − 7.15_sex_; *p*-values < 0.05), 12.10 for reading comprehension (88.91 + 0.22_euthymia_–_trait_ − 4.92_age_; *p*-values < 0.01), 6.59 for problem-solving (21.95 + 0.16_euthymia_–_trait_ + 10.53_educational stage_; *p*-values < 0.05), and 11.90 for number and calculation (70.40 + 0.16_euthymia_–_trait_ + 16.23_educational stage_ − 6.79_sex_; all *p*-values < 0.05, except for sex, *p* = 0.063).

## 4. Discussion

This study aimed to analyze the relationships between sleep, depression, and cognitive performance in adolescents. The results revealed significant bidirectional relationships between adolescents’ sleep (daytime sleepiness and sleep quality) and depression (state and trait), and between their depressive symptoms and performance in inductive reasoning, reading comprehension, and mathematical thinking. According to our results, adolescents’ depression-trait, or their personal disposition to suffer from depressive symptoms in general, is more related to their cognitive performance than to depression-state, or the depressive symptoms that they might be suffering from at a particular time. Even more important, the results point to the relevant role of euthymia above dysthymia when it comes to predicting their cognitive performance. However, neither daytime sleepiness nor sleep quality seems to necessarily affect cognitive performance in adolescents, as the results do not show any significant association between these two variables and any cognitive domain. In contrast, some sociodemographic variables (i.e., sex and age or educational stage) might impact cognitive performance in this population. Although no significant differences were directly found between boys and girls in cognitive performance, they did show these differences in sleep and depression; and sex proved to be a significant predictive factor for adolescents’ performance in some domains. Age or educational stage also affected the results, with younger participants sleeping better and older participants showing better cognitive performance.

Interestingly, our results regarding euthymia as a positive predictor of cognitive performance in adolescents above dysthymia seem to be in line with the new way of understanding this construct in recent years. Specifically, though euthymia was firstly conceived as the absence of mood disturbances or depressive symptoms, some authors proposed a novel conception of this construct that involved both the absence of mental illness and the presence of positive emotions or subjective well-being [50]. This approach has been further developed and explored in subsequent studies, indicating the importance of reinforcing this construct—beyond reducing dysthymia—for the clinical practice in terms of promoting mental health and protecting against depression [51,52,53,54]. Herein fits our study, in which the presence of positive emotions—or the disposition to experience positive emotions—was shown to be more related to cognitive performance than the presence of—or tendency to experience—negative emotions or depressive symptoms did. In other words, according to our results, not only the absence of depressive symptoms would be a good indicator of better cognitive performance, but the presence of positive emotions would also be necessary to predict such performance, thus understanding both conditions as a continuum.

Following the aforementioned approach, it is also convenient to bear in mind the link of euthymia with psychological well-being and resilience [50] when explaining our findings. It should particularly be borne in mind because some results of preceding studies also point to a positive association between psychological well-being or resilience and cognitive or executive functioning in different age ranges (e.g., [55,56,57,58]). This was contrary to what was observed, partially, in relation to sleep. In this regard, the associations of daytime sleepiness and sleep quality with euthymia and dysthymia found in this study are undeniable in light of the well-documented relationship between disrupted sleep and depression (see [59], for meta-analytic evidence). However, our results regarding the relationship between sleep and cognitive performance seem to be opposite to those reported in prior research.

As some studies highlighted an association between sleep problems and cognitive impairment (see [60], for a review), one would expect to find some significant correlations between daytime sleepiness or sleep quality and cognitive performance. Nevertheless, our failure to find significant associations between sleep-related variables and cognitive performance might have been due to the absence of sleep disorders among our participants, as the presence of such disorders was precisely an exclusion criterion. In contrast, it is not always possible to rule out this possibility in other studies in which such eligible criterion was not applied, or significant sleep problems were directly reported by participants (e.g., [39]). The fact that the association between sleep and cognitive performance could be particularly strong in people with sleep disorders compared to good sleepers, could be a plausible explanation for our findings. Another explanation might be the focus on subjective sleep measures, as discrepancies between subjective and objective sleep measures are common in research (e.g., [61,62]), and the relationship between sleep and cognitive performance could be different depending on the sleep parameter taken into account. Indeed, this possible reason was already suggested by the authors of a recent study, after the results did not reveal any significant association between cognitive performance and sleep quality in healthy young adults [63].

In relation to the potential impact of some sociodemographic variables on adolescents’ cognitive performance, the higher daytime sleepiness and lower sleep quality reported by girls agree on the higher prevalence of insomnia recently estimated among females than in males [64]. Similarly, according to available evidence, females are more prone to suffer from depression than males do [65,66]; therefore, it is not strange that girls showed a lower level of euthymia and a higher level of dysthymia than boys did in this study. Concerning the effect of age or educational stage, the fact that cognitive performance was better in older participants (third- and fourth-year students), regardless of other variables, might be mainly due to two reasons. The first is the evolutionary development itself; that is, the maturation (emotional) processes that occur at these ages. This conclusion coincides with other previous studies [67,68]. The second is at the end of compulsory secondary education when students define their own curricular development (preferably in the fourth year). Moreover, other aspects more related to the teaching-learning process itself and, specifically, to strategies and competencies, may be influencing the cognitive domains (see [69,70]. Furthermore, it should be remembered that compulsory secondary education means a change of educational stage, which very often implies a shift from school to high school. This leads to a loss of significant affective ties and the need for creating a new circle of friends which, in short, translates into an important change in the lives of adolescents. Therefore, it would be reasonable to assume that first- and second-year students were more likely to experience adjustment problems that also affected their cognitive function, thus impacting on our results.

Some existing limitations in this study should be borne in mind when interpreting its results and drawing conclusions. The most important one relates to the representativeness of the sample, as it would be desirable to check whether there were variations in the results depending on different school settings, regions, or ethnic groups. The inclusion of other variables linked to mental health—beyond depressive symptomatology—such as anxiety symptoms or lifestyle habits (i.e., diet and physical exercise) could also contribute to a better understanding of cognitive functioning in adolescents. Similarly, the relationship between sleep, depression, and cognitive functioning was examined by considering performance in some specific cognitive domains. The consideration of a larger set of performance scores, both cognitive and academic, would help to test if the relationships found between those variables remained across different cognitive skills or they even impacted on school marks. It cannot be ignored either that the data were collected using self-reports, which implies the analysis of sleep with a single focus on subjective sleep measures. An assessment of sleep also using polysomnography or actigraphy would allow contrasting objective and subjective sleep parameters at the time of analyzing the results.

Beyond the aforementioned limitations, the findings of this study expand knowledge about a topic of traditional importance and even more since the COVID-19 lockdown. As highlighted in the introduction, despite the growing interest in the factors linked to cognitive performance in adolescents, a considerable number of studies focused on adults or elderly people [5,6,7,8,9,10,11], or on some specific populations (i.e., people with some disorders [12,13,14,15,16,17]). Consequently, our study contributes to extending previous research on this topic by providing data on typically developing adolescents and considering two important factors, apart from some sociodemographic variables: sleep and depression. In this regard, it would be convenient to recall that depression constitutes one of the most incidental and prevalent mental diseases in adolescents worldwide [18,19].

## 5. Conclusions

The results of this study might suggest that sleep does not always affect cognitive performance in adolescence, and point to significant relationships between the latter and depression, thus highlighting the role of euthymia—beyond dysthymia—on predicting cognitive function at this developmental stage. It would emphasize the need for promoting positive emotions without aiming to decrease the negative ones only as a way of improving cognitive performance in adolescents, also taking into account their age and sex, and the educational stages in which they are. Nevertheless, further studies should analyze these relationships in larger and different samples and should consider paying attention to other variables potentially related to sleep, depression, and cognitive functioning, such as anxiety symptoms, diet, and exercise. Forthcoming studies could also explore the interrelationships between all these variables and examine the differences, depending on sociodemographic factors, to determine adolescents’ performance in other cognitive domains or in academic achievement.

## Figures and Tables

**Table 1:** Correlation coefficients between sleep, depression, and cognitive performance variables.

	V1	V2	V3	V4	V5	V6	V7	V8	V9	V10	V11	V12	V13
**V1**	−												
**V2**	0.16 *	−											
**V3**	0.18 **	0.21 **	−										
**V4**	−0.10	−0.17 **	−0.18 **	−									
**V5**	0.10	0.20 **	0.33 ****	−0.26 ****	−								
**V6**	−0.03	−0.08	−0.07	0.86 ****	0.15 *	−							
**V7**	−0.03	−0.01	−0.18 **	0.64 ****	−0.25 ***	0.53 ****	−						
**V8**	0.06	0.24 ***	0.34 ****	−0.36 ****	0.55 ****	−0.11	−0.30 ****	−					
**V9**	−0.02	0.13	−0.03	0.44 ****	0.02	0.51 ****	0.82 ****	0.14 *	−				
**V10**	−0.15 *	−0.07	0.07	0.11	−0.06	0.07	0.23 ***	−0.14 *	0.14 *	−			
**V11**	−0.22 ***	−0.06	0.01	0.10	−0.20 **	−0.01	0.22 **	−0.17 *	0.13 *	0.61 ****	−		
**V12**	−0.04	−0.04	0.04	0.01	−0.02	0.02	0.16 *	−0.10	0.10	0.57 ****	0.47 ****	−	
**V13**	0.06	−0.01	0.08	0.02	−0.11	−0.01	0.16 *	−0.13 *	0.08	0.61 ****	0.43 ****	0.59 ****	−

Abbreviations: V1 = age; V2 = daytime sleepiness; V3 = sleep quality; V4 = euthymia-state; V5 = dysthymia-state; V6 = state total score; V7 = euthymia-trait; V8 = dysthymia-trait; V9 = trait total score; V10 = inductive reasoning; V11 = reading comprehension; V12 = problem-solving; V13 = number and calculation. * *p* < 0.05, ** *p* < 0.01, *** *p* < 0.001, **** *p* < 0.0001.

**Table 2:** Students’ sleep, depression, and cognitive measures depending on sex.

Measure	Girls ^1^	Boys ^1^	*W*	*p*	Effect Size *r*
Daytime sleepiness	8.32 (4.06)	7.18 (4.41)	8543.5	0.029	−0.14
Sleep quality ^2^	5.57 (8.08)	4.16 (2.59)	8355.0	0.037	−0.13
Dysthymia-state	24.21 (28.67)	32.32 (33.53)	6341.0	0.093	−0.11
Euthymia-state	74.91 (24.89)	85.42 (18.30)	4991.5	<0.001	−0.26
State total score	65.20 (17.43)	77.39 (17.44)	3918.5	<0.001	−0.39
Dysthymia-trait	20.15 (26.52)	23.56 (29.46)	7028.0	0.808	−0.02
Euthymia-trait	71.22 (27.17)	82.06 (21.65)	5331.0	<0.001	−0.22
Trait total score	58.74 (21.38)	68.26 (18.99)	5216.5	<0.001	−0.23
Inductive reasoning	39.67 (24.88)	35.70 (27.59)	7668.5	0.136	−0.10
Reading comprehension	36.62 (23.52)	35.70 (25.46)	7102.0	0.602	−0.03
Problem-solving	39.09 (24.51)	40.03 (25.00)	6333.0	0.723	−0.02
Number and calculation	43.77 (26.88)	38.39 (25.95)	7416.5	0.122	−0.10

^1^ Mean (SD). ^2^ Higher score means lower sleep quality.

**Table 3:** Students’ sleep, depression, cognitive measures, and post-hoc pairwise comparisons per academic year.

Measure	1 ^1^	1 vs. 2 ^3^	1 vs. 3 ^3^	1 vs. 4 ^3^	2 ^1^	2 vs. 3 ^3^	2 vs. 4 ^3^	3 ^1^	3 vs. 4 ^3^	4 ^1^	*H*	*p*
Daytime sleepiness	6.75 (4.49)	2570.00 (0.03)	2584.00 * (0.21)	2194.00 * (0.22)	6.85 (4.12)	3666.50 * (0.02)	3150.50 * (0.20)	8.47 (3.78)	5240.00 (0.02)	8.70 (4.52)	10.00	0.019
Sleep quality ^2^	3.51 (2.48)	2289.00 (0.14)	2363.50 ** (0.28)	1977.50 ** (0.32)	4.05 (2.20)	3653.00 (0.16)	3076.00 * (0.19)	4.94 (2.59)	5145.50 (0.06)	6.89 (11.70)	14.65	0.002
Dysthymia-state	25.60 (30.61)	--	--	--	22.82 (27.93)	--	--	30.21 (31.88)	--	32.68 (33.98)	3.23	0.357
Euthymia-state	86.40 (18.66)	3066.00 (0.12)	4193.00 * (0.22)	2618.00 ** (0.25)	79.47 (23.63)	4881.50 (0.08)	3146.50 (0.11)	80.08 (20.69)	3645.50 (0.06)	74.47 (25.87)	8.08	0.045
State total score	74.29 (20.47)	--	--	--	69.05 (21.06)	--	--	70.81 (16.88)	--	70.26 (15.51)	3.83	0.280
Dysthymia-trait	19.08 (29.47)	--	--	--	19.41 (24.97)	--	--	24.59 (28.48)	--	22.67 (29.05)	3.66	0.300
Euthymia-trait	79.92 (23.57)	--	--	--	74.51 (24.60)	--	--	76.74 (26.96)	--	74.12 (25.62)	4.80	0.187
Trait total score	65.54 (24.20)	--	--	--	60.56 (18.45)	--	--	64.08 (21.66)	--	62.59 (19.16)	3.52	0.318
Inductive reasoning	40.8 (32.96)	--	--	--	36.85 (25.63)	--	--	36.88 (22.33)	--	37.47 (26.29)	0.14	0.986
Reading comprehension	41.67 (25.47)	--	--	--	34.95 (25.86)	--	--	35.37 (20.81)	--	34.11 (26.46)	3.25	0.355
Problem-solving	35.05 (26.74)	2984.00 (0.00)	2055.00 * (0,30)	2429.00 (0.07)	33.71 (19.86)	2896.00 *** (0.39)	2758.50 (0.07)	51.49 (21.7)	2438.00 *** (0.39)	32.52 (26.1)	28.24	<0.001
Number and calculation	32.49 (25.39)	2317.50 (0.01)	2044.50 *** (0.34)	1887.00 * (0.23)	33.88 (25.54)	3123.00 *** (0.31)	2897.50 (0.18)	50.47 (26.02)	3081.00 (0.12)	43.71 (25.15)	19.80	<0.001

^1^ Mean (SD). ^2^ Higher score means lower sleep quality. ^3^ Wilcoxon rank-sum (effect size *r*). Abbreviations: 1 = 1st year; 2 = 2nd year; 3 = 3rd year; 4 = 4th year. * *p* < 0.05, ** *p* < 0.01, *** *p* < 0.001.

## Data Availability

Data available on request.
